# Efficacy and safety of praziquantel preventive chemotherapy in *Schistosoma mansoni* infected school children in Southern Ethiopia: A prospective cohort study

**DOI:** 10.3389/fphar.2023.968106

**Published:** 2023-03-01

**Authors:** Tigist Dires Gebreyesus, Eyasu Makonnen, Tafesse Tadele, Kalkidan Mekete, Habtamu Gashaw, Heran Gerba, Eleni Aklillu

**Affiliations:** ^1^ Department of Global Public Health, Karolinska Institutet, Stockholm, Sweden; ^2^ Ethiopian Food and Drug Authority, Addis Ababa, Ethiopia; ^3^ Center for Innovative Drug Development and Therapeutic Trials for Africa, College of Health Sciences, Addis Ababa University, Addis Ababa, Ethiopia; ^4^ Departments of Pharmacology and Clinical Pharmacy, College of Health Sciences, Addis Ababa University, Addis Ababa, Ethiopia; ^5^ College of Medicine and Health Sciences, Hawassa University, Hawassa, Ethiopia; ^6^ Ethiopian Public Health Institute, Addis Ababa, Ethiopia

**Keywords:** Efficacy and safety, praziquantel, schistosomiasis, cure rate, egg reduction rate, preventive chemotherapy, deworming, pharmacovigilance

## Abstract

**Background:** The World Health Organization recommends efficacy and safety surveillance of anti-helminths used in mass drug administration campaigns. We evaluated the effectiveness of single-dose praziquantel against *Schistosoma mansoni* infection, and the safety of praziquantel plus albendazole preventive chemotherapy (PC) in *Schistosoma mansoni* infected school children (n = 512) in Southern Ethiopia.

**Method:** Stool examinations were done using thick smear Kato-Katz at baseline, week-4, and week-8 of post-Mass drug administration (MDA) to assess praziquantel efficacy. Participants were followed for MDA-associated adverse events up to day 7 of post-MDA. The primary and secondary study outcomes were praziquantel efficacy (parasitological cure and egg reduction rates) and MDA-associated adverse events (AEs), respectively.

**Result:** The overall cure rates at week-4 and week-8 were 89.1% (95%CI = 86.1–91.7) and 87.5% (95%CI = 83.6–90.8), respectively. Cure rates among moderate-to-heavily infected children were significantly lower (*p* = 0.001) compared to those with light infection at week-4 (84.4% vs. 91.1%, *p* = 0.03) and week-8 (78.6% vs. 91.9%, respectively). Older children had a higher cure rate than younger ones at week-8 (90.1% vs. 79.5%, *p* = 0.01). Among those who were *Schistosoma* egg-free (cured) at week 4, 7.8% became egg-positive at week 8. The overall egg reduction rate (ERR) at week-4 and week-8 were 93.5% and 91.3%, respectively, being lower among the 5–9 years old age groups (*p* = 0.01) at week-8. The proportion of children who remained *schistosoma* egg-positive throughout the study follow-up period was 4.6%, and their ERR at week-4 and week-8 was 50% and 51%, respectively, which is below the 90% World Health Organization threshold for efficacy. The incidence of experiencing at least one type of MDA-associated AEs were 17.0% (95%CI = 13.8%–20.5%); abdominal pain, headache, and vomiting were the most common. The proportion of mild, moderate, and severe AEs was 63.2%, 26.3%, and 10.5%, respectively. Females experienced more AEs than males (*p* = 0.03).

**Conclusion:** Single-dose praziquantel is still effective for the treatment of intestinal schistosomiasis. Praziquantel and albendazole preventive chemotherapy is safe and tolerable, and associated AEs are mostly mild-to-moderate and transient. However, the reduced PZQ effectiveness in moderate-to-heavy infection and observed AEs in about one-fifth of infected children underscores the need for better treatment strategies and surveillance for early detection of parasite resistance and management of AEs.

## Introduction

The World Health Organization (WHO) currently recognizes 20 neglected tropical diseases (NTDs) mainly occurring in the tropics and sub-tropics (WHO, 2010). Schistosomiasis, the second most prevalent NTD, causes a significant public health problem in many parts of the world, mainly affecting people in Africa, Asia, the Caribbean, and South America (Hotez et al., 2007; U Olveda, 2013; Vos et al., 2017). *Schistosoma haematobium* (*Schistosoma haematobium*) and *S. mansoni* (*Schistosoma mansoni*) are the two most common species affecting people living in Sub-Saharan Africa (SSA), where 90% of the global disease burden occurs (Hotez and Kamath, 2009; Mnkugwe et al., 2020b; Gebreyesus et al., 2020). Schistosomiasis mainly affects children, and its chronic infection can lead to various health complications, including anemia, malnutrition, impaired childhood development, poor cognitive function, fatigue, and exercise intolerance (King and Dangerfield-Cha, 2008; Ezeamama et al., 2018).

Large-scale preventive chemotherapy with periodic mass administration of single-dose PZQ to all at-risk populations in endemic settings (deworming) is the core strategy to control and eliminate schistosomiasis recommended by the WHO. Previously, WHO set a target to eliminate schistosomiasis as a public health problem by reducing the disease burden to less than 1% with heavy infection by 2025 (WHO, 2013b). In addition to preventive chemotherapy, the WHO recommends supplementary control measures such as the provision of safe and clean drinking water, basic sanitation and hygiene, vector (snail) control, and health education (WHO, 2013b, 2015a). The revised WHO roadmap for NTD targets the elimination of schistosomiasis as a public health problem and interrupts schistosome transmission in endemic countries by 2030 (WHO, 2022a), contributing towards achieving Sustainable Development Goal 3.

During the past 2 decades, millions of school-age children received periodic PZQ preventive chemotherapy annually in endemic areas with a prevalence rate of ≥50% infection. The recent WHO recommendation increased the target population for PZQ preventive chemotherapy to all at risk age groups from 2 years old with 75% treatment coverage in areas where the prevalence of schistosomiasis infection is ≥10%. This scale-up in target population for preventive chemotherapy may impose a potential risk for parasite resistance (WHO, 2022b). The potential emergence of anthelmintic resistance could endanger progress towards these ambitious goals due to repeated sub-optimal drug exposure. Therefore, continued vigilance of the efficacy and safety of PZQ is recommended to evaluate the impact of the intervention and detect any reduced performance which may occurred due to parasite resistance (WHO, 2013a; WHO, 2022b). The world relies on PZQ for the treatment and prevention of schistosomiasis since there are no alternative drugs approved for the treatment of schistosomiasis so far (WHO, 2013a). Therefore, monitoring the treatment effectiveness is crucial as previous studies reported suboptimal PZQ efficacy (egg reduction rate <90%) and decreased cure rate after treatment (Crellen et al., 2016; Vale et al., 2017).

Repeated use of PZQ, especially in high transmission areas, may result in reduced efficacy (Crellen et al., 2016). Despite the repeated use of PZQ, the prevalence of schistosomiasis remains high in some areas (Alebie et al., 2014; Alemayehu and Tomass, 2015). Studies from SSA reported a low cure rate after single-dose PZQ administration for the treatment of schistosomiasis (Raso et al., 2004; Garba et al., 2013; Nalugwa et al., 2015; Crellen et al., 2016). Due to the reported low cure rate and reduced efficacy of PZQ after repeated administration in some areas, the WHO recommends that the NTD programs implementing MDA of PZQ to incorporate regular efficacy assessment in their working standard to monitor and evaluate the efficacy of the drug (WHO, 2010, 2015a). To sustain the impact of preventive chemotherapy and avoid possible future parasite resistance, additional measures like availing adequate clean water, implementing hygiene and sanitation programs, educating the community, and exploring other treatment options are crucial (Mnkugwe et al., 2020a).

*Schistosoma mansoni,* which causes the intestinal form of schistosomiasis, is endemic throughout Ethiopia (WHO_Africa, 2016). More than one-third of the Ethiopian population (39 million) is currently living in schistosomiasis endemic area (Leta et al., 2020), and the disease remains among the leading causes of morbidity in the country (Jember, 2014). Since 2014, the national NTD program of Ethiopia has been implementing targeted school-based mass drug administration (MDA) with PZQ and ALB annually in regions with a moderate-to-high prevalence of schistosomiasis and STHs infections (WHO_Africa, 2016). Although MDA is currently expanded to all endemic areas, surveillance data on treatment outcomes is limited. The efficacy and safety surveillance during PZQ mass drug administration (MDA), especially in high transmission areas, is essential to inform policymakers and NTD control programs on the effectiveness of the intervention in curing the diseases and reducing transmission. This study evaluated the efficacy of single-dose PZQ in treating *S. mansoni* infection and the safety of PZQ plus ALB preventive chemotherapy in *S. mansoni* infected school children in Ethiopia.

## Methodology

### Ethical statement

The study received ethical approval from Southern Nations, Nationalities, and People’s region Health Bureau ethical review committee (Ref no 902-6-19/14966) and the National Research Ethics Review Committee (Ref no MoSHE//RD/141/9848/20). Permission to conduct the study was obtained from regional, zonal, and woreda Health and Education offices. In addition, training for laboratory professionals and awareness creation meetings with relevant stakeholders were held to provide information about the study, including the study objective, significance, procedures, and type of data to collect. Before enrolment, participants and their parents/legal guardians received information about the study. For participants ≤12 years old, written informed consent was obtained from their parents/guardians. For participants >12 years of age, written informed consent was obtained from the parents/guardians, and assent was obtained from the study participants.

### Study design, area, and population

This prospective observational PZQ efficacy and safety of PZQ and ALB co-administration surveillance study was conducted from January to March 2019. The study objective, design, and protocols used were in accordance with the WHO recommendations for assessing the efficacy of PZQ against schistosomiasis in *S. mansoni-*infected children (WHO, 2013a), and to conduct safety surveillance of drug combinations used in preventive chemotherapy interventions for the control of NTDs (WHO, 2011).

The study population was *S. mansoni* infected school children (5–15 years old) attending four primary schools in two rural districts (Hawella Tula and Wondo Gennet) in Southern Ethiopia. The two study districts were classified as high-prevalence endemic areas for *S. mansoni* according to the national mapping for schistosomiasis and soil-transmitted helminths (Leta et al., 2020).

Regardless of infection status, all children attending the four primary schools were eligible to receive preventive chemotherapy containing PZQ and ALB provided through the national Ethiopian NTD public health program. Two weeks before MDA, stool samples were collected at baseline for microscopic examination for screening and diagnosis of *S. mansoni* infection. Only *S. mansoni-*infected school children were enrolled for PZQ efficacy against *S. mansoni* infection and safety of PZQ plus ALB co-administration.

### Data collection procedures

Baseline socio-demographic data such as age, sex, weight, and height measurements were collected using the case record format prepared for the study. Anthropometric measurements were converted to Z-score for assessing stunting and wasting (HAZ = height for age) and BMI for age (BAZ) using WHO anthro-plus software version 1.0.4 (WHO, 2015b). Participants with less than two standard deviations for HAZ and BAZ scores were considered stunted and wasted.

### Diagnosis, treatment, and follow-up

The WHO preventive chemotherapy or deworming guideline recommends co-administration of PZQ and ALB tablets against schistosomiasis and STHs, respectively. Therefore, the current study assessed the efficacy of praziquantel against intestinal schistosomiasis in *S. mansoni* infected children. However, the safety assessment concerns both PZQ and ALB since the two drugs were co-administered as part of preventive chemotherapy to all children attending the selected schools as scheduled by the Ethiopian national NTD control program, Ministry of Health. The study team had no role in the MDA planning or administration of the drugs.

Fresh stool samples were collected 2 weeks before MDA for screening and diagnosis of *S. mansoni* infection using Kato-Katz technique. A follow-up stool sample was collected from infected children at the fourth and eighth weeks of post-treatment for assessment of PZQ efficacy (cure rate and egg reduction rates). Double thick Kato-Katz smears were prepared from each stool sample using 41.7 mg templates, and the two slides were read by different laboratory professionals to determine the *S. mansoni* egg counts (Katz et al., 1971). The slides were read after keeping them for hours to ensure clear visibility of *Schistosoma* eggs. Quality assurance was performed on 10% of the slides by independent senior laboratory professionals. The mean egg count for each participant was calculated as an average of the two slides egg count. The eggs per gram of stool (epg) were calculated by multiplying the mean egg count by a factor of 24 (WHO, 1991). The intensity of infection was categorized as light (1–99 epg), moderate (100–399 epg) and heavy (≥400 epg) according to the WHO criteria (WHO, 2013b).

The study participants received a single dose of PZQ and ALB as part of preventive chemotherapy through school-based campaigns led by the district health office of the NTD control program. The number of PZQ tablets was calculated based on the height of the children using the WHO dose pole (≥94 cm dose pole to deliver a dose of at least 40 mg/kg) (Montresor et al., 2005). In addition, one tablet of ALB 400 mg was co-administered to all children regardless of age, weight or height following the national NTD program and the WHO MDA guideline.

### Study outcomes and definitions

The primary study outcome was PZQ treatment effectiveness (parasitological cure and egg reduction rates at four- and 8 weeks post-treatment). The cure rate was defined as the proportion of treated children who were egg-positive before MDA but became egg-negative at week four and week eight after treatment. The egg reduction rate (ERR) was calculated (WHO, 2013a) as follows:

$$ERR=\left[1-\left(arithmetic\;mean\;of\;epg\;after\;treatment/arithmetic\;mean\;of\;epg\;before\;treatment\right)\right]\times 100$$

The secondary study outcome was the incidence and type of MDA-associated adverse events (post-MDA AEs), defined as any event that was not reported before treatment but occurred during the 7-follow-up period after MDA. Before MDA, study participants were interviewed for any pre-existing clinical symptoms (pre-MDA event) such as fever, loss of appetite, dizziness or fainting, confusion, drowsiness, headache, cough, difficulty in breathing, nausea, vomiting, diarrhea, stomach pain, itching, rash, and any other symptoms. Study participants were actively and prospectively monitored for the occurrence of any AEs on day 1, and on day 7 of post-MDA. Between days 2–6, participants reported if they experienced any AEs. The severity grading of observed post-MDA AEs was done following Common Terminology Criteria for Adverse Events (CTCAE) version 5.0 (NIH, National cancer institute, 2017).

### Statistical analysis

Data collected for the study was initially entered into an Excel sheet, then cleaned and exported to Statistical Package for Social Sciences (SPSS) software for Windows version 24 (SPSS, IBM Corp, Armonk, NY, United States) for analysis. Descriptive statistics were used to summarize the baseline socio-demographic characteristics of study participants. The Chi-square test was used to compare the test differences between groups of independent variables. Mann Whitney *U* test was used to compare the difference in mean ranks of ERR from baseline and post-MDA between groups. Univariate and multivariate regressions were used to identify predictors of cure rate. Variables with a *p* < 0.2 in univariate were included in the multivariate model. A *p-value* < 0.05 was considered statistically significant.

## Result

A total of 512 *S. mansoni*-infected children were enrolled in this study and received single-dose PZQ and ALB as preventive chemotherapy. Safety data were collected from all study participants during the 7-follow-up period. PZQ efficacy data were obtained from 507 to 345 children at weeks four and eight follow-up visits, respectively. The socio-demographic characteristics of the study participants are presented in Table 1. The frequency distributions of male and female participants were almost similar. At baseline, most participants had light infection intensity (71%) followed by moderate (24.1%). About 59.4% of the study participants were co-infected with STH. The proportions of wasted and stunted children were 8.7% and 16.4%, respectively.

**TABLE 1:** Baseline socio-demographic characteristics of study participants.

Variable	Category	Week 4	
		Number	%
Age group	5–9	112	22.1
	10–15	395	77.9
Sex	Male	253	49.9
	Female	254	50.1
Intensity of infection	Light	360	71
	Moderate	122	24.1
	Heavy	25	4.9
Kebele/Village	Tula	272	53.7
	Finchawa	18	3.6
	Wosha	217	42.8
Schools	Bushulo	132	26
	KidusPawulos	140	27.6
	Finchawa	18	3.6
	Wosha	217	42.8
District	Hawella Tula	290	57.2
	WondoGennet	217	42.8
HAZ	Stunted	83	16.4
BAZ	Wasted	44	8.7
STHs co-infection	Yes	301	59.4
	No	206	40.6

### Parasitological cure rates and associated factors

**FIGURE 1:**
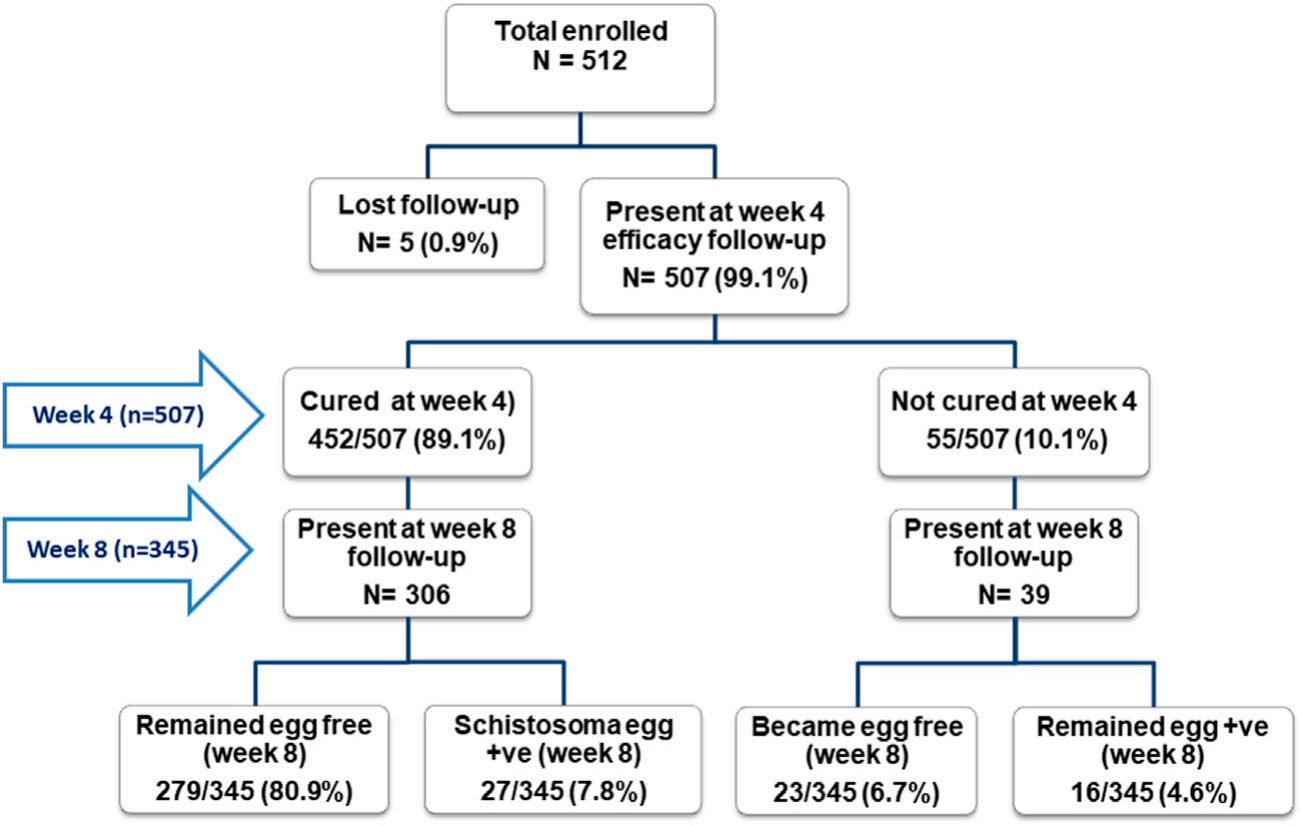
Study flow chart and observed cure rates stratified by treatment follow-up visits (in weeks).

Study enrollment and flow chart with cure rate stratified by follow-up visits for the efficacy study (week four and week eight post-MDA) is presented in Figure 1. Cure rates (CR) and egg reduction rates (ERR) data were collected from 507 to 345 children at week four and week 8, respectively. The overall cure rates at week four and week eight of post-MDA were 89.1% (452/507, 95%CI = 86.1–91.7) and 87.5% (302/345, 95%CI = 83.6–90.8), respectively. The cure rates at week four significantly varied among the baseline infection intensity categories. Children with moderate to heavy infection intensity at baseline had significantly lower cure rates than those with light infection at both study follow-up weeks [week 4; 84.4% *versus* 91.1%, *p* = 0.026, Odds ratio (OR) = 1.90 (95% CI of OR = 1.05–3.33), at week 8 (78.6% *versus* 91.9%, *p* = 0.001, OR = 2.80 (95% CI of OR = 1.49–5.25)] (Figure 1; Table 2). Similarly, the cure rate was significantly higher (*p* = 0.01) among the older age groups (90.1%) compared to the lower age groups (79.5%) at week 8. Older age groups were more cured (90.1%) compared to the younger age groups (85.7%) at week 4 (*p* = 0.18). The cure rate was significantly associated with age group (*p = 0.01*) and baseline infection intensity (*p = 0.001*) at week eight follow-up. The cure rate was higher among male participants (90.1%) compared to females (88.2%) (Table 2).

**TABLE 2:** Cure rate and associated factors at week four and week eight of post-treatment.

Variables		Week 4 (n = 507)				Week 8 (n = 345)			
		Cured N (%)	Not cured N (%)	X^2^	*p*	Cured N (%)	Not cured N (%)	X^2^	*p*
Overall		452 (89.2)	55 (10.8)			302 (87.5)	43 (12.5)		
Infection intensity	Light	328 (91.1)	32 (8.9)	4.9	**0.026**	225 (91.9)	22 (8.9)	10.1	**0.001**
	Moderate-to-heavy	124 (84.4)	23 (15.6)			77 (78.6)	21 (21.4)		
Age group	5–9	96 (85.7)	16 (14.3)	1.76	0.18	66 (79.5)	17 (20.5)	6.44	**0.01**
	10–15	356 (90.1)	39 (9.9)			236 (90.1)	26 (9.9)		
Sex	Male	228 (90.1)	25 (9.9)	0.49	0.48	152 (87.9)	21 (12.1)	0.03	0.86
	Female	224 (88.2)	30 (10.8)			150 (87.2)	22 (12.8)		
Schools	Bushulo	117 (88.6)	15 (11.4)	3.61	0.31	84 (90.3)	9 (9.7)	3.63	0.30
	Finchawa	18 (100)	0 0)			9 (100)	0 0)		
	Kiduspawulos	121 (86.4)	19 (13.6)			103 (83.7)	20 (16.3)		
	Wosha	196 (90.3)	21 (9.7)			106 (88.3)	14 (11.7)		
Stunting (HAZ)	Stunted	72 (86.8)	11 (13.2)	0.59	0.44	47 (88.7)	6 (11.3)	0.07	0.78
	Not stunted	380 (89.6)	44 (10.4)			255 (87.3)	37 (12.7)		
Wasting (BAZ)	Wasted	41 (93.2)	3 (6.8)	0.81	0.37	28 (82.4)	6 (17.6)	0.92	0.33
	Not wasted	411 (88.8)	52 11.2)			274 (88.1)	37 (11.9)		
STH co-infection	Yes	268 (89.0)	33 (11.0)	0.01	0.92	169 (87.1)	25 (12.9)	0.07	0.79
	No	184 (89.3)	22 (10.7)			133 (88.1)	18 (11.9)		

Significant values are indicated in bold.

On the follow-up visit, 7.8% (27/345) of the children who were cured (*Schistosoma* egg-free) at week four became egg positive at week 8 (Figure 1). Sixteen children (4.6%, 16/345) were persistently not cured during the study period (egg-positive both at week four and week 8). Among the 16 children who failed to cure, 44% (7/16) had moderate to heavy infection intensity before treatment.

To identify predictors of cure rate at week 4, and week 8, we conducted univariate followed by multivariate logistic regression analysis. In the multivariate regression, baseline infection intensity was the only significant predictor of cure rate, being lower in moderate-to-heavy infected participants than those with light infection at baseline (*p* = 0.026). At week 8, the lower age group and moderate-to-heavy infection intensity at baseline were significant independent predictors of lower cure rate (*p* = 0.002 and 0.012) (Table 3).

**TABLE 3:** Predictors of cure rate at weeks four and week eight of post single dose praziquantel administrations.

Variables		Cured N (%)	Week 4				Week 8			
			Univariate		Multivariate		Univariate		Multivariate	
			cOR (95% CI)	*p*	aOR (95% CI)	*p*	cOR (95% CI)	*p*	aOR (95% CI)	*p*
Intensity of infection	Light	328 (91.1)								
	Moderate-to-Heavy	124 (84.4)	0.53 (0.3–0.9)	0.028	0.5 (0.3–0.9)	**0.028**	0.4 (0.2–0.7)	0.002	0.4 (0.2–0.7)	**0.002**
Age group	5–9	96 (85.7)	0.7 (0.4–1.2)	0.18	0.7 (0.3–1.2)	*0.18*	0.4 (0.2–0.8)	0.013	0.4 (0.2–0.8)	**0.012**
	10–15	356 (90.1)								
Sex	Male	228 (90.1)	0.8 (0.5–1.4)	0.48			0.99 (0.5–1.9)	0.98		
	Female	224 (88.2)								
STH infection	Yes	268 (89.0)								
	No	184 (89.3)	1 (0.6–1.8)	0.92			1.1 (0.6–2.1)	0.68		
HAZ	Normal	411 (88.8)								
	Stunted	41 (93.2)	1.3 (0.7–2.7)	0.44			1.3 (0.7–2.4)	0.442		
BAZ	Normal	380 (89.6)								
	Wasted	72 (86.8)	0.6 (0.2–1.9)	0.37			0.6 (0.2–1.9)	0.372		

COR, crude odd ratio; AOR, Adjusted odd ratio; CI, Confidence Interval; Significant values are indicated in bold.

### Egg reduction rates

The overall egg reduction rates at weeks four and week eight follow-up visits were 93.7% and 91.3%, respectively. The egg reduction rate was higher among participants with baseline moderate-to-heavy infection intensity compared to the light ones (88.3% vs. 95.7% at week 4 and 89.6% vs. 91.9% at week 8) at both follow-up visits (Table 4). The ERR among the lower age groups was much lower than the older age groups (ERR = 82.7% *versus* 94.1%) at week eight follow-up. Interestingly, the egg reduction rate among the 16 children who were persistently not cured throughout the study period was 50% and 51% at week four and week 8, respectively.

**TABLE 4:** The Arithmetic mean egg count at baseline and follow up visits with their respective ERR stratified by baseline characteristics.

Variables	Category	Baseline line egg count (Mean + SD)	*p¥*	Week 4 egg count (Mean + SD)	*p¥*	ERR^a^ (%) week 4	Week 8 egg count (Mean + SD)	*p¥*	ERR* (%) week 8
Age group	5–9	111.4 + 150.2	0.51	8.12 + 34.9	0.20	92.7	19.32 + 53.4	0.01	82.7
	10–15	112.4 + 173.7		7.01 + 37.7		93.8	6.68 + 34.6		94.1
Sex	Male	105.6 + 168.9	0.08	4.52 + 18.8	0.47	95.7	10.68 + 43.9	0.92	89.9
	Female	120.8 + 176.4		9.73 + 47.9		91.9	7.88 + 34.3		93.5
Baseline infection intensity	Light	43.5 + 25.0	<0.001	5.1 + 23.9	0.03	88.3	4.51 + 19.9	<0.001	89.6
	Moderate-to-heavy	283.4 + 246.0		12.2 + 56.3		95.7	21.43 + 65.4		91.9
STH Co-infection	No Co-infection	106.8 + 137.3	0.02	5.77 + 27.3	0.82	94.6	6.95 + 29.9	0.61	93.5
	Co-infected	117.4 + 193.5		8.07 + 41.7		93.1	11.13 + 45.4		90.5

a ERR, 100 [1 − (Arithmetic mean epg after treatment/Arithmetic mean epg before treatment)], ¥: Mann Whitney *U* test.

### Treatment-associated adverse events

Eighty-seven participants out of 512 reported a total of 152 adverse events. The proportions of mild, moderate, and severe adverse events were 63.2%, 26.3%, and 10.5%, respectively. Most (77%) adverse events occurred on the first day of MDA and were resolved by day 7 of MDA. The incidence of experiencing at least one type of MDA-associated adverse event during the 7-day follow-up was 17.0% (87/512, 95% CI = 13.8%–20.5%). The five most common adverse events were abdominal pain (13.3%), headache (5.7%), vomiting (3.5%), nausea (1.8%), and diarrhea (1.6%), respectively. The proportion of study participants who experienced one, two, and ≥ three types of adverse events were 50.6%, 29.9%, and 19.5%, respectively. Sex was the only significant factor associated with post-MDA adverse events (*p* = 0.03), where female participants experienced more adverse events. Though not statistically significant, the proportion of participants who experienced adverse events was higher in heavy infection intensity (24.0%) than the mild (16.6%) and moderately (16.8%) infected individuals at baseline. Table 5 summarizes factors associated with MDA-related adverse events.

**TABLE 5:** Factors associated with occurrence of adverse events.

Variables	Category	Adverse events		X^2^	*p*
		No (N/%)	Yes (N/%)		
Sex	Male	231 (86.5)	36 (13.5)	4.87	0.03
	Female	194 (79.2)	51 (20.8)		
Age Category	5–9 years	90 (85.7)	15 (14.3)	0.69	0.41
	10–15 years	335 (82.3)	72 (17.7)		
HAZ	Normal	338 (83.3)	68 (16.7)	0.08	0.77
	Stunted	87 (82.1)	19 (17.9)		
BAZ	Normal	381 (82.8)	79 (17.2)	0.10	0.75
	Wasted	44 (84.6)	8 (15.4)		
Intensity of Infection	Mild	312 (83.4)	62 (16.6)	0.17	0.68
	Moderate-to-Heavy	113 (81.9)	25 (18.1)		
Number of PZQ tablet taken	Less than 3 tablets	302 (84.1)	57 (15.9)	1.05	0.30
	≥3 tablets	123 (80.4)	30 (19.6)		

## Discussion

We investigated the effectiveness of single-dose PZQ for the treatment of *S. mansoni* infection and the safety of PZQ plus ALB preventive chemotherapy among infected schoolchildren in southern Ethiopia. PZQ efficacy was assessed using the cure rate and egg reduction rate at week four and week eight to rule out any possible re-infection after being cured. Our result showed an overall high cure rate of PZQ with 89.1% and 87.5% at week four and week 8, respectively. Pre-treatment infection intensity and age were significant predictors of the cure rate. The observed egg reduction rate of 93.7% and 91.3% at week-4 and week-8, respectively, is above the 90% efficacy threshold set by WHO (WHO, 2013a). Accordingly, single-dose PZQ resulted in a satisfactory egg reduction rate at week four and week eight follow-ups. However, a reduced cure rate was observed among moderate to heavily infected and lower age group infected children. The higher egg reduction rate in moderate-to-heavy infected individuals may indicate that PZQ is still effective in reducing disease morbidity. However, preventive chemotherapy alone may not be adequate for controlling and eliminating schistosomiasis because the reduced cure rate in young children and moderate-to-heavily infected individuals may impede the progress toward halting the transmission.

The overall cure rate observed in our study at week four and week eight follow-up visits was nearly equal to the upper margin of the documented standard cure rate (60%–90%) for single-dose PZQ treatment (WHO, Expert committee report, 2002). This indicates that single-dose PZQ treatment resulted in satisfactory cure rate among school children in our study. Although PZQ preventive chemotherapy successfully reduced morbidity, PZQ alone may not be sufficient for the control and elimination of schistosomiasis, partly due to its poor efficacy against juvenile schistosomes (Inobaya et al., 2014). Despite the observed high cure rates in this study, 7.8% of the participants who were cured at week four became egg positive at the week eight follow-up visit. This might be attributed to either re-infection (new infection) or reduced egg clearance due to the poor PZQ effectiveness against juvenile worms, which may attain reproductive maturity to start laying eggs after week 4 (Inobaya et al., 2014). This could show the inadequacy of single-dose PZQ preventive chemotherapy alone to prevent and control *S. mansoni*. WHO also recommends the integration of other interventions in addition to the MDA towards preventing re-infection (WHO, 2022a). Repeated doses of PZQ or combining it with other anthelmintics that kills the juvenile schistosome is recommended to improve the efficacy against schistosomiasis (Munisi et al., 2017; Bergquist and Elmorshedy, 2018). A recent randomized clinical trial reported superior efficacy of PZQ and Dihydroartemisinin-piperaquine combination therapy (targeting both the adult and juvenile worm, respectively) than PZQ alone for treating intestinal schistosomiasis (Mnkugwe et al., 2020a).

The cure rate significantly varied across the infection intensity groups at baseline. The cure rate among moderate-to heavily infected children was significantly lower than children who had light infection intensity. In addition, the cure rate was lower among the younger age groups. This could be explained by the fact that the older age groups received higher doses of PZQ since the drug was given based on height. Like the cure rate, the ERR in the younger children was much lower than in older children at week-8 follow-up (Tables 2 and 4). A similar result was reported in a recent systematic review (Fukushige et al., 2021). Therefore, younger age groups may be at increased risk of reduced efficacy due to low cure rate and ERR.

The overall egg reduction rates observed at week four and week eight were above the 90% ERR threshold recommended by WHO guideline for assessing the efficacy of anthelmintic drugs (WHO, 2013a) which indicates its satisfactory result. Contrary to the lower cure rate, the moderate-to-heavily infected individuals had the highest ERR at both follow-up periods. A previous study reported that PZQ has a larger impact on the parasite fecundity, which may result in low egg count post-treatment (Lamberton et al., 2017). This may explain the higher ERR in moderate-to-heavily infected individuals. Since the morbidity of schistosomiasis is related to the burden of egg intensity (Fukushige et al., 2021), higher ERR among the moderate-to-heavily infected individual may reduce the morbidity associated with the disease and hence halt the transmission. Preventive chemotherapy in the form of MDA in endemic areas is implemented with the primary objective of morbidity reduction by reducing the intensity of infection after treatment (Kabatereine et al., 2003). Likewise, this higher egg reduction rate observed shows that single-dose PZQ treatment reduces morbidities associated with high egg burden in infected children which eventually help in the prevention of transmission for meeting the elimination goal.

PZQ and ALB were generally tolerated in our study participants, though 17% of the participants experienced at least one MDA-associated AE. Previous studies on school children reported an even higher incidence of AEs than we observed (Erko et al., 2012; Zwang and Olliaro, 2017; Muthoni et al., 2018). The relatively lower incidence of AEs could be due to our rigorous definition set for MDA-associated AEs (i.e., pre-existing clinical symptoms were cross-checked to verify that the same type of event was not reported after MDA). Higher incidence of MDA-associated AEs among females than males was observed, which is in line with recent studies conducted in SSA countries which assessed the safety of MDA for lymphatic filariasis (Khaemba et al., 2021; Fimbo et al., 2022). Like previous reports, abdominal pain and nausea were the most common AEs in our study. Most of the reported AEs were mild to moderate and transient, which indicates that single-dose PZQ and ALB treatment is safe and tolerable. However, the observed severe AEs in some children highlight the need to integrate safety surveillance into the MDA campaigns. Although millions of children are receiving periodic MDA in Africa, safety data are scarce partly due to the region’s lack of fully functional pharmacovigilance systems (Barry et al., 2020; 2021).

To assess PZQ efficacy, we used the Kato-Katz techniques as recommended by the WHO guideline, which indicates the CR and ERR reference thresholds to conclude on drug effectiveness (WHO, 2013a). Kato-Katz method is reported to be less sensitive in low infection intensity compared to the urine-based point-of-care circulating cathodic antigen test (POC-CCA). The WHO does not recommend detecting CCA using the POC-CCA test for parasitological drug efficacy assessment (WHO, 2013a). The POC-CCA test is recommended for mapping schistosomiasis prevalence and post-treatment infection monitoring (WHO, 2013b; Straily, et al., 2021). The suitability of the POC-CCA test in monitoring drug efficacy requires further evaluation, including the precise time for CCA clearance following PZQ treatment in different age groups and epidemiological setting remains to be established (Mazigo et al., 2021; Yin et al., 2021). Although the Kato-Katz technique is highly specific and can accurately detect high-intensity infection, its decreased sensitivity for diagnosis may result in overestimating the efficacy observed in our study, hence our study limitation. The Kato-Katz method used in our study may have a minor impact on our findings since the study was conducted in a high-infection-intensity area (Gebreyesus et al., 2020). Nevertheless, this PZQ efficacy and the safety surveillance of mass PZQ and ALB co-administration conducted in high transmission areas of Ethiopia may provide data to policymakers and NTD control programs on the effectiveness of the intervention in curing the diseases and reducing transmission.

## Conclusion

In summary, single-dose PZQ treatment is still effective for treating *S. mansoni* infection and in reducing morbidity among moderate-to-heavy infected children, as shown by significant ERR. However, the observed lower cure rate, failure to cure, and re-infection in moderate-to-heavily infected and younger children may indicate reduced efficacy of PZQ. Therefore, preventive chemotherapy with PZQ alone may not be adequate to halt transmission and ultimately eliminate schistosomiasis as a public health problem. Alternative treatment strategies such as repeated dosage or combination therapy targeting juvenile and adult worms and integrating other preventive measures are recommended. Overall preventive chemotherapy with PZQ and ALB is generally safe and tolerable, and one-in-five schistosomiasis-infected children experience mild to moderate and transient adverse events. Our study finding highlights the need to integrate periodic efficacy and safety surveillance in the MDA programs for early detection of parasite resistance and management of AEs.

## Data Availability

The original contributions presented in the study are included in the article/supplementary material, further inquiries can be directed to the corresponding author.
